# COVID-19 risk, attitudes and behaviour study (CRAB study): A knowledge, attitudes, and practise qualitative study of COVID-19 in the Royal Navy

**DOI:** 10.3389/fpubh.2022.1101817

**Published:** 2023-01-12

**Authors:** Stephen D. Woolley, Robert Chambers, Jonathan R. B. Bishop, Amy Logan, Peter McMillan, Thomas E. Fletcher, Miriam Taegtmeyer, Matthew K. O'Shea

**Affiliations:** ^1^Department of Clinical Sciences, Liverpool School of Tropical Medicine, Liverpool, United Kingdom; ^2^Institute of Naval Medicine, Alverstoke, United Kingdom; ^3^Tropical and Infectious Diseases Unit, Liverpool University Hospitals Foundation NHS Trust, Liverpool, United Kingdom; ^4^Royal Navy Healthcare, Royal Navy Headquarters, HMS EXCELLENT, Portsmouth, United Kingdom; ^5^NIHR SRMRC, University Hospitals Birmingham NHS Foundation Trust, Birmingham, United Kingdom; ^6^Academic Department of Military Medicine, Royal Centre for Defence Medicine, Joint Hospital Group, ICT Building, Birmingham Research Park, Birmingham, United Kingdom; ^7^Department of International Public Health, Liverpool School of Tropical Medicine, Liverpool, United Kingdom; ^8^Institute of Immunology and Immunotherapy, College of Medical and Dental Sciences, University of Birmingham, Birmingham, United Kingdom

**Keywords:** COVID-19, Navy, survey, military, vaccine hesitancy

## Abstract

**Introduction:**

Outbreaks of SARS-CoV-2 onboard maritime platforms spread rapidly and have high attack rates. The aim of the COVID-19 Risk, Attitudes and Behaviour (CRAB) study was to investigate the knowledge, attitudes, and practises in the Royal Navy in relation to COVID-19 prevention.

**Methods:**

The CRAB study was a cross-sectional survey, using a census sampling method, conducted in May and June 2021. An online questionnaire was distributed to all serving Royal Navy regular personnel using either the MyNavy application or *via* a QR code through email for a continuous 14 day period. The questionnaire was based on an existing validated questionnaire used for avian influenza epidemics. Questions investigated individual perceptions of COVID-19 seriousness, compliance with prevention methods, explored vaccination intention and vaccine hesitancy (unvaccinated individuals who declined or were unsure about receiving a COVID-19 vaccine). The chi-squared test of best fit was used to compare the demographic responses against the whole organisation, with *p*-value < 0.05 deemed significant. Odds ratios were used to investigate associations between demographic groups and responses to questions, with an odds ratio crossing 1.0 deemed non-significant.

**Results:**

The response rate was 6% (2,080/33,200), with 315 responses collated in the pilot phase and 1,765 in the main study phase. Male participants were less likely to rate COVID-19 as serious (OR 0.34; 95% CI: 0.23–0.49). BAME ethnicity (OR 2.41; 95% CI: 1.12–5.17) rated it as more serious. At the time of the study 62% of respondents had received one dose of a COVID-19 vaccine. In the 797 unvaccinated personnel, vaccine hesitancy accounted for 24.2% (193/797), of whom 136 were white males. Those who had a higher COVID-19 serious rating, the most significant factor for non-adherence to COVID-19 prevention measures in both vaccinated (OR 1.61 [95%CI: 1.20–2.17]) and vaccine-hesitant (OR 3.24 [95%CI: 1.63–6.41]) individuals was colleagues' non-adherence. The most trusted source of information on vaccines was provided by the Defence Medical Services (77.2% [1,606/2,080]).

**Conclusion:**

This study has identified reasons for COVID-19 protective measure adherence, sources of information trusted by respondents and vaccine hesitancy, in the Royal Navy. The questionnaire can be used to investigate attitudes and behaviours in future emerging infectious diseases.

## 1. Introduction

The first reported cases of COVID-19 diagnosed in the United Kingdom (UK) was on 27 January 2020 (1) and the World Health Organisation (WHO) declared COVID-19 a global pandemic on 11 March 2020 (2). It is well recognised that viral respiratory infections have high attack rates onboard maritime platforms (3–5) as documented in early civilian and military outbreaks on ships (6–9). The Royal Navy quickly adopted a quarantine and isolation policy consisting of 14-day isolation in single ensuite accommodation and SARS-CoV-2 polymerase chain reaction (PCR) testing on day 0, 7, and 12. This policy was able to mitigate some of the risk of exposure and onward transmission, although once the virus was onboard a vessel, large outbreaks were typically observed (6–9).

Early control measures were largely based in identifying and isolating contacts as well as reducing social mixing through lockdown measures. The four nations of the UK went into a full lockdown, with only essential movement of people, on 3 occasions, with the last lockdown in England ending fully by 19 July 2021 (10). The compliance with National Health Service (NHS) Test and Trace and COVID-19 lockdowns was unknown in UK Armed Forces personnel during the COVID-19 pandemic. Adherence to the NHS Test and Trace has been reported at 18.2% between March and September 2020 (11), with up to 75% of household contacts of positive cases leaving home (11). The drop in compliance to NHS Test and Trace quarantine, was observed just as national guidance for self-isolation for testing positive to SARS-CoV-2 was reduced from 14- to 10-days (10).

Previous studies regarding the SARS-CoV-2 virus and associated risk-taking behaviours and attitudes have been conducted among civilian populations, investigating factors leading to an increase in preventative behaviours during the early phase of the pandemic. Increasing age, higher educational attainment, female sex and perceived fear have been identified the most protective factors (11–15). Socio-economic factors that appear to appear to demonstrate better compliance to COVID-19 preventative behaviours are education and occupation (13, 15). Higher education status particularly linked to higher knowledge of the disease and therefore better compliance (14). Those with higher education are also likely to be in more senior occupational roles (14) and therefore consider not just the impact of the disease on them but also their work. Those individuals who are compliant are most likely to be compliant to all the guidelines, rather than just some (14). Older age were also more likely to adopt COVID-19 compliance measures (15).

Prior to this study, little was known about these factors among military personnel. In general, risk-taking behaviour in UK Armed Forces personnel, who are predominantly male and younger age is variable, with increases in impulsive-sensation seeking behaviour in combat arms, especially when controlled for age and gender (16, 17).

The UK was one of the first nations to licence rapidly developed SARS-CoV-2 vaccinations and to implement a national vaccination programme, which started in December 2020 (18). UK Armed Forces personnel were voluntarily vaccinated through the national programme, with older and higher risk populations vaccinated first. Individuals were provided with the same information about the safety and efficacy of the vaccines as the civilian UK population, as well specific information endorsed by the UK Surgeon General which further detailed military-specific information regarding the safety, efficacy, occupational risks, deployability, and vaccination rollout. Individual members of the military were given time to book and attend their vaccination through NHS vaccination centres, with transportation arranged for those in remote locations or unable to travel independently. The national vaccination rollout for adults below the age of 40 years occurred concurrently with the CRAB study.

Conflicting social media messaging, such as misinformation about vaccine-induced infertility, reduced confidence in COVID-19 vaccine safety which is reported to have increased vaccine hesitancy in younger populations (19). The 5C model is one of several models developed to predict vaccine intention and behaviours (20). The five components of this model consist of: Confidence (trust and effectiveness of vaccines), Complacency (perceived risk and threat of vaccine-preventable diseases), Constraints [Convenience] (psychological and physical barriers to vaccination), Calculation (individual data gathering), and Collective Responsibility (individual willingness to protect others by getting vaccinated) (20). The 5C model is applicable to military populations due to factors affecting the key predictors such as geographical availability of vaccines, potential direct and indirect restrictions on data gathering and collective responsibility to protect colleagues.

The aim of the CRAB study was to investigate the knowledge of COVID-19, attitudes to COVID-19 preventative measures, motivations to comply with disease control measures, information requirements regarding COVID-19 and attitudes towards COVID-19 vaccination, with further subgroup analysis by demographics, among serving Royal Navy (RN) personnel. We hypothesised that older age, those in a more senior rank, female sex and BAME ethnicity were most likely to adhere to COVID-19 guidelines and have high vaccine uptake.

## 2. Materials and methods

### 2.1. Study design and participants

**Figure 1:**
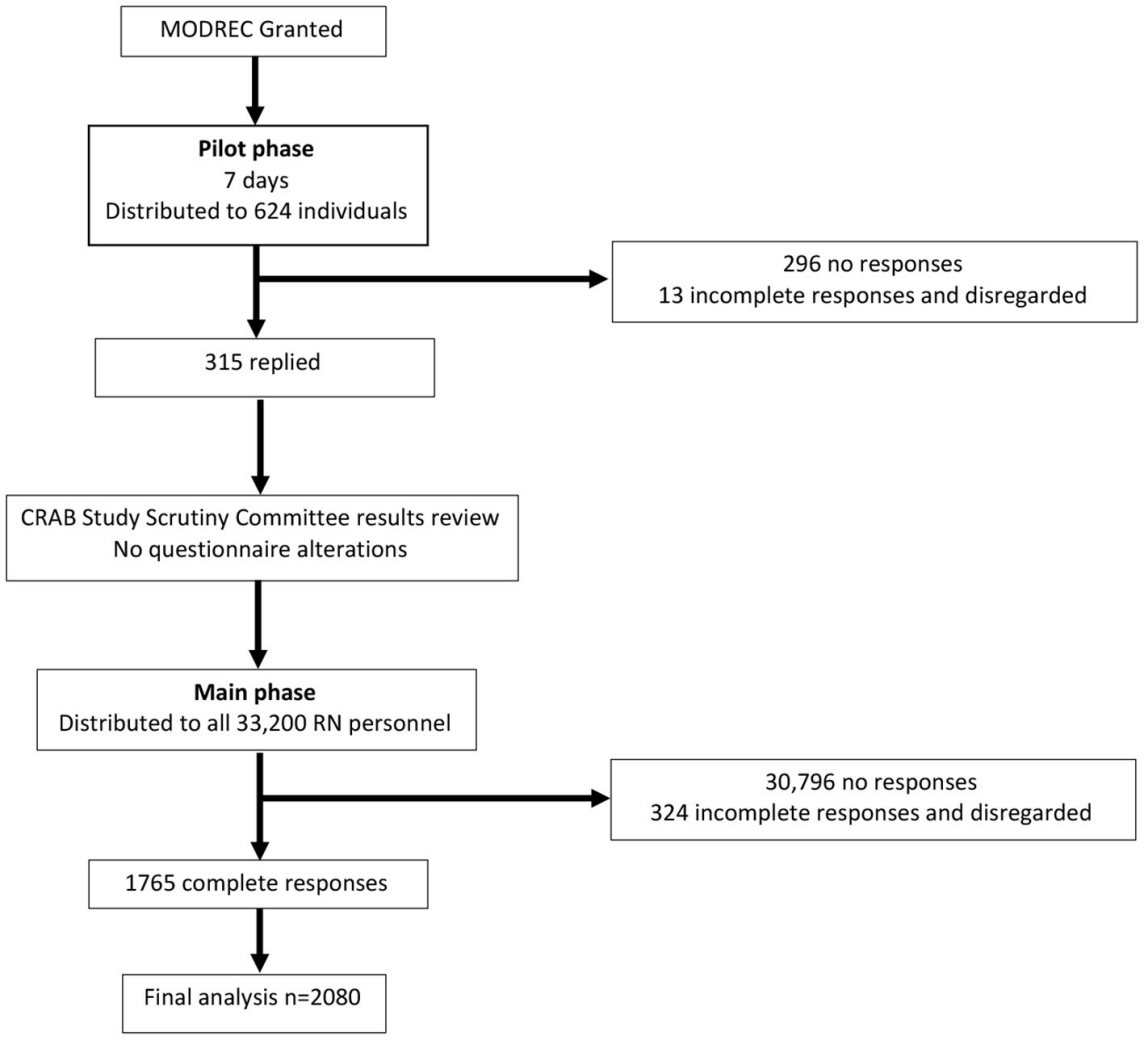
Schema of study. n, number; MODREC, Ministry of Defence Research Ethics Committee.

The study used a cross-sectional design and administered an online questionnaire in two phases, consisting of a small pilot phase (17 May to 24 May 2021) followed by the main study phase (24 May to 7 June 2021) (Figure 1). The study was conducted as a census sample, taking a “snapshot” of the whole RN organisation of ~33,000 serving personnel, with an anticipated response rate of 20–25% based on previous studies (12). The proposed response rate was based on the return rate by rank rather than age alone, as returns in lower age groups are considered to be lower than older populations.

The questionnaire was conducted online using the Lime Survey application. Participants accessed the Lime Survey, *via* the MyNavy application or QR codes distributed to each naval/marine shore establishment and afloat unit. Every member of the RN has their own MyNavy account, and approved recruitment messages were distributed *via* the MyNavy administration team. On opening the link, participant information detailed the aims of the study and outlined the voluntary nature and anonymity. The questionnaire was configured not to store any personal information. The pilot phase was opened for 7 days on the 17 May 2021, with over 100 participants asked to reply, from a cross-section of the total study population. Results were scrutinised by the CRAB study steering group for any inconsistencies in responses. The main phase of the study was launched on the 24 May 2021 and remained open for 14 days. A preliminary report of key findings was produced and distributed to the senior RN leadership to assist in policy formation (Figure 1).

### 2.2. Questionnaire

The questionnaire was based on the Effective Communication in Outbreak Management (ECOM) tool (21), initially designed to assess attitudes and behaviours towards 2009 H1N1 pandemic avian influenza in European urban and ethnic minority groups. The ECOM tool consists of 35 questions using a mix of Likert scale and best-answer questions (21). The ECOM questionnaire was designed following expert panel review, demonstrating good convergent validity (*r* = 0.86), although reliability was not formally assessed, however it did undergo a pilot phase (*n* = 29) and five think-aloud-interviews leading to minor modifications (18). This questionnaire was chosen as the basis of the CRAB questionnaire due to its design. COVID-19 specific questions were added and exiting questions modified. We replaced the “unnamed disease” in the ECOM questionnaire with COVID-19, with the questions modified to compare COVID-19 against other infectious diseases such as influenza and meningitis. Questions were grouped into five areas: knowledge of COVID-19; attitudes to COVID-19 preventative measures; motivations to comply with disease control measures; information requirements regarding COVID-19 and attitudes towards COVID-19 vaccination (Supplementary Figure 1).

Demographic data included age, sex (male/female), rank, ethnicity, and educational attainment. Age was grouped into four categories (16–24, 25–34, 35–44, and >45 years). Ranks were categorised using North Atlantic Treaty Organisation (NATO) rank ranges: R1–R4 (junior ranks), R6–9 (non-commissioned officers [NCO]), OF1–OF3 (junior officers), and OF4+ (senior officers). Ethnicity was based on Census 2021 groupings. Educational attainment was recorded according to UK educational framework levels: Level 2 (GCSEs and Scottish Nationals), Level 3 (A-levels and Scottish Highers), and Level 5 onwards (Bachelor's degree or higher). The RN branch was divided into warfare, Royal Marines (RM), logistics, medical, engineers, aircrew, and others (e.g., chaplains, training management officers and other smaller branches not previously included).

To improve uptake and reduce responder bias, the questionnaire was configured to take < 10 min to complete, used non-leading questions which were short and easily interpreted. The more controversial questions were included at the end. The questionnaire was reviewed by two clinical psychologists with substantial experience of questionnaire design.

### 2.3. Ethics

Full ethical approval was obtained from the Ministry of Defence Research Ethics Committee (2031/MODREC/21).

### 2.4. Statistical analysis

Data were cleaned using frequency lists to identify invalid characters and missing values. The pilot and main study data were combined for cleaning and analysis. Data were analysed using Stata v17.1 (StataCorp LLC, Texas, US) and R Statistical Software (v3.6.1, R Core Team 2019). Descriptive statistics were used to compare demographics, with medians and interquartile ranges (IQR) used after normality testing. The chi-square goodness of fit test was used to test significance (*p* < 0.05) of the observed proportions against the proportions across the whole organisation. Unadjusted ordinal logistic regression models were used to explore the relationship between demographics (age, sex, ethnicity, rank, educational attainment, and branch) and knowledge of COVID-19, attitudes to COVID-19 preventative measures, motivations to comply with disease control measures, information requirements regarding COVID-19 and attitudes towards COVID-19 vaccination. These are reported as odds ratios (OR) with 95% confidence intervals (CI) with an odds ratio crossing 1.0 deemed non-significant.

## 3. Results

### 3.1. Responses and demographics

The total number of responses were 2,080 from 33,200 personnel (response rate of 6.3%). The 16–24- and 35–44-year-old groups were the highest responders (*n* = 564/2,080 and *n* = 565/2,080, respectively, 27%), although that did not match the total proportions in those sub-groups across the whole organisation using chi-squared test of best fit (*p* < 0.001) (Table 1). 1,721 (83%) respondents were male which was a lower proportion than across the total organisation using chi-squared test of best fit (*p* < 0.001). 1,978/2,080 respondents (95%) identified as White (English, Welsh, Scottish, Northern Irish, or British), which represents a similar proportion across the Royal Navy using chi-squared test of best fit (*p* = 0.84). According to the 2020 UK Armed Forces biannual diversity statistics 91% of the Royal Navy workforce was male, with 4.6% from a BAME ethnicity (22). By rank, the largest group of responders was the junior rank cohort (*n* = 877/2,080, 42%), which was lower than the proportion across the organisation (*p* < 0.001). The largest cohort by highest educational attainment was Level 5 (*n* = 769, 37%), followed by the Level 3 (*n* = 673/2,080, 32%).

**Table 1:** Summary of demographic responses across the organisation.

		**No. of survey respondents (*n*)**	**Percentage of total survey respondents (%)**	**No. across RN (*n*)**	**Percentage of the total RN (%)**	***P*-value**
Age	16–24	565	27	9,080	27	
	25–34	489	23	12,260	37	
	35–44	565	27	8,100	25	
	45+	461	22	3,760	11	
	Total	2,080		33,200		**< 0.001**
Biological sex	Male	1,721	83	29,980	90	
	Female	359	17	3,220	10	
	Total	2,080		33,200		**< 0.001**
Ethnicity	White	1,978	95	31,710	95	
	BAME	89	4	1,490	5	
	Prefer not to say	13				
	Total	2,080		33,200		0.84
Rank	R1–R4 (Junior rank)	877	42	18,140	54	
	R6–R9 (NCO)	541	26	8,495	26	
	OF1 to OF3	481	23	4,579	14	
	OF4+	181	9	1,986	6	
	Total	2,080		33,200		**< 0.001**
Educational attainment*	GCSE or equivalent (Level 2)	638	31			
	A-levels or equivalent (Level 3)	673	32			
	Bachelor's degree or higher (Level 5+)	769	37			
	Total	2,080		33,200		

n, number; BAME, black and minority ethnic groups; R, other ranks; RN, Royal Navy; OF, officer. ^*^An accurate total across the organisation is not available. Test of significance using Chi-square goodness of fit comparing the observed responses compared to proportions across the whole Royal Navy.

The bold values indicated to illustrate significant values to a reader quickly reading the article.

### 3.2. Knowledge of COVID-19

Most participants (1,548/2,080, 74.4%) rated meningitis as serious (5/6) or extremely serious (6/6), compared to 43.5% (905/2,080) for COVID-19 and 26.9% (560/2,080) for influenza. When considering the level of concern about becoming infected with COVID-19 over the next 12 months, 27.7% of individuals (576/2,080) were somewhat concerned about being infected with COVID-19 with 4.9% (102/2,080) who were very concerned and 22.9% (477/2,080) not concerned at all. Of those who were not concerned, 65% (310/477) were aged ≤ 35 years and 55% (263/477) were junior ranks (R1–4).

Overall knowledge of COVID-19 symptoms and transmission was high. The majority of responders (2,038/2,080, 98%) understood COVID-19 may be asymptomic, that COVID-19 can be acquired more than once (1,914/2,080, 92%) and that there is a vaccine offering protection from COVID-19 (1,934/2,080, 93%). Subgroup analysis showed that among responders aged ≤ 35 years 97.8% (1,030/1,054) knew COVID-19 could be asymptomatic, 98.2% (1,034/1,054) knew COVID-19 can be acquired more than once, and 92.0% (968/1,054) knew there was COVID-19 vaccine offering protection. Among white males under the age of 35 years (*n* = 796), 1.9% (15/796) thought COVID-19 was only a symptomatic disease, 1.8% (14/796) thought it could only be contracted once and 93.5% (744/796) understood there was a vaccine available. Among this group who did not believe there was a vaccine against COVID-19 (52/796), there were very different perceptions of the seriousness of COVID-19 when compared to influenza and meningitis (Supplementary Figure 1).

### 3.3. Attitudes to force health protection measures and compliance

When questioned about force health protection measures (FHPM), especially non-pharmaceutical interventions (NPIs) such as facemask wearing, social distancing and regular testing using lateral flow devices (LFDs), many individuals (1,707/2,080, 82.1%), felt isolation of positive and suspected cases reduced the risk of COVID-19, with face coverings being considered the least effective measure (1,429/2,080, 68.7%). Among white male responders who had not received a dose of a COVID-19 vaccine, were unsure or not going to receive a dose (*n* = 136), most identified isolation of positive cases (41/136, 30.1%) and frequent cleaning (29/136, 21.3%) as the most certain ways to reduce the risk of acquiring COVID-19 (Supplementary Figure 2).

The single greatest motivation to adhere to COVID-19 FHPM was the protection of family (942/2,080, 45.3%), followed by the protection of colleagues (474/2,080, 22.8%). Those who did not want to affect the functioning of their unit and were vaccine hesitant did not perceive COVID-19 to be as serious as those who were vaccinated (OR 0.57 [95%CI: 0.34–0.96]), whereas the most significant factor to motivate NPI adherence in those vaccinated compared to those unvaccinated was concern about being ill as they deemed COVID-19 to be more serious (OR 1.96 [95%CI: 1.47–2.60]).

Factors associated with non-adherence to COVID-19 FHPM were regular LFD testing (819/2,080, 39.4%), and COVID-19 vaccinations (763/2,080, 36.7%). In those who considered COVID-19 to be more serious, the most significant factor for non-adherence to COVID-19 NPIs in both vaccinated (OR 1.61 [95%CI: 1.20–2.17]) and vaccine-hesitant (OR 3.24 [95%CI: 1.63–6.41]) responders was work colleagues' non-adherence to the same FHPM when compared to those who thought measures didn't work. Those who had been vaccinated deemed COVID-19 to be less serious when going to see family (OR 0.69 [95%CI: 0.54–0.88]) and friends (OR 0.62 [95%CI: 0.48–0.82]) when compared to those unvaccinated.

### 3.4. Information regarding COVID-19

Most individuals trusted their respective medical centre or the Defence Medical Services in providing information regarding the COVID-19 vaccinations (1,606/2,080, 77.2%), followed by UK Government websites (1,573/2,080, 75.6%). Responders considered that religious leaders (22/2,080, 1.1%) and social media (40/2,080, 1.9%) were the least trusted information sources. Information regarding the COVID-19 vaccine (836/2,080, 40.1%) was the most popular topic for requesting further information.

### 3.5. Intention to vaccinate against COVID-19

Just under two thirds of the study population had received one dose of a COVID-19 vaccine in the first 6 months of the national vaccine rollout (1290/2,080, 62%). Of the remaining 797 participants, 193 (24.2%) showed vaccine hesitancy, declining the vaccine or were unsure about receiving it. Analysis of the perceived seriousness by both age and vaccine hesitancy showed that among participants in the 16–24-year age group who did not intend to consent to be vaccinated against COVID-19,' 12% (12/98) rated the seriousness of being infected by COVID-19 as a 1 (not at all serious). In the 25–34 age group more than 25% (*n* = 15/61) rated the seriousness of being infected by COVID-19 as a 1 (not at all serious).

Ordinal regression modelling showed that those who identified their ethnicity as BAME rated COVID-19 as more serious, compared to those who identified as White (OR 2.41 [95%CI: 1.12-5.17]). Male participants considered COVID-19 less serious than females (OR 0.34 [95%CI: 0.23–0.49]). The senior NCOs and junior officers viewed COVID-19 as less serious than junior ranks (OR 0.57 [95% CI: 0.39–0.85] and 0.53 [0.38–0.75], respectively). Similarly, those who were in logistics or “Other” branch considered COVID-19 more serious than those in the warfare branch. Responders with a maximum educational attainment of GCSEs or equivalent considered COVID-19 more serious, when compared to those with a bachelor's degree or higher (OR 1.72 [95%CI: 1.23–2.41]) (Table 2).

**Table 2:** Association between COVID-19 severity score and vaccine hesitancy by demographics.

		**No. of respondents (*n*)**	**Odds ratio**	**95% CI**
Age group	16–24	565	1	NA
	25–34	489	0.76	0.58–1.01
	35–44	565	0.91	0.63–1.33
	45+	461	0.78	0.32–1.94
Biological sex	Female	359	1	NA
	**Male**	**1,721**	**0.34**	**0.23–0.49**
Ethnicity	White	1,978	1	NA
	**BAME**	**89**	**2.41**	**1.12–5.17**
	Preferred not to say	13	0.38	0.06–2.55
Rank	R1–OR4 (Junior rank)	877	1	NA
	**R6–OR9 (NCO)**	**541**	**0.57**	**0.39–0.85**
	**OF1–OF3**	**481**	**0.53**	**0.38–0.75**
	OF4+	181	0.59	0.13–2.58
Role/Branch	Warfare	536	1	NA
	Royal Marines	188	0.6	0.32–1.11
	**Logistics**	**242**	**2.41**	**1.54–3.78**
	Medical	237	1.56	0.64–3.84
	Engineer	667	0.98	0.72–1.35
	Air Crew	72	1.11	0.57–2.15
	**Other**	**137**	**2.29**	**1.36–3.86**
Education	Bachelor's degree or above	638	1	NA
	A/AS level (or equivalent)	673	1.06	0.76–1.48
	**GCSEs—any grade (or equivalent)**	**769**	**1.72**	**1.23–2.41**

Summary of ordinal logistic regression model investigating the association between COVID-19 severity score and vaccine hesitancy by demographics. The demographic and vaccine hesitancy status are categorical predictors with the COVID-19 seriousness the ordinal outcome. BAME, black and minority ethnic group; OR, other rank; OF, officer.

The bold values indicated to illustrate significant values to a reader quickly reading the article.

## 4. Discussion

The CRAB study is the first knowledge, attitudes and practise survey regarding COVID-19 in a UK military population, and the first to survey a whole military department, with other surveys only assessing small sub-groups of a service (23–25). Knowledge of COVID-19 transmission and symptoms was high across those who were surveyed, although after 15 months of the pandemic, the seriousness of the disease was considered comparable to influenza. Isolation of positive cases and frequent cleaning were the two NPIs thought to be the most likely to prevent COVID-19, with protection of family and colleagues the two primary factors for respondents to adhere to NPIs. As vaccination numbers increased, COVID-19 was judged to be less serious in those vaccinated and a strong factor for non-adherence to NPIs. Those identified as vaccine hesitant appear comparable to rates among the UK civilian population, despite being a military population at increased risk due to exposure in constrained populations onboard military vessels (19).

The Royal Navy predominantly comprises of white Caucasian males, and the CRAB study is one of the largest surveys of this demographic but also adds valuable data to previous surveys and questionnaires targeting female, BAME and immunosuppressed populations (26–28). The 5C vaccination intention model is a useful tool to assess attitudes and behaviours to vaccines (20). In this study there was a high “confidence” in the vaccines (19). The perception that COVID-19 mostly affects older populations may engender “complacency” and therefore may contribute to vaccine hesitancy in this population (29). “Collective responsibility” may be a factor in increasing vaccine uptake in military populations. While not explicitly investigated in this study, previous studies have shown it to be a strong predictor of why individuals would adhere to COVID-19 NPIs (30). The Royal Navy did not place any barriers preventing individuals from being vaccinated, similar to their civilian counterparts in the national vaccination rollout programme, therefore “constraints” is unlikely to be a significant reason for vaccine hesitancy. The request for more information regarding the vaccines appears to be comparable to other groups, although the trust with government and internal medical sources appears higher than previously observed (19).

Female sex, BAME ethnicity, being part of the logistics or other branch of the RN or having a maximal educational attainment of GCSEs or equivalent were associated with a higher COVID-19 seriousness score. Whilst age was not significant in determining COVID-19 seriousness, it is likely the junior ranks are younger, reflecting that age may have an impact. This study identified those who were older considered COVID-19 to be less serious. This is in contrast to data which suggests that older age is associated with higher compliance with preventative behaviours (11).

There were several limitations to this study. The study was only available for 14 days in the main phase; therefore, the response rate was low at 6.2% and below the intended response rate of 20–25%. The study window was 14 days, due to the haste of the UK vaccination rollout. If there was more time, and due to the low response rate, a stratification sampling method would have been the next step. The census sampling method was employed to take a rapid and easier “snapshot” of the organisation. As such, our findings may not reflect the wider RN population and therefore not generalisable for the whole organisation, although there were 2,000 responses. The data produced in the pilot phase was scrutinised by the study team for major discrepancies, although formal statistical testing of survey reliability and validity were not conducted, however it was based on a previously validated questionnaire. With a small sample size, the study is at risk of responder bias, although some mitigation measures were undertaken including the use of short concise questions, use of non-leading questions, use of interval questions and a short survey completion time. The largest cohort in this study were educated to degree level or higher. Higher educational attainment appeared to be associated with decreased compliance with preventative measures (31). While several vaccine hesitant individuals were identified, further information based on the 5C model was not collected, especially around confidence (vaccine safety and efficacy concerns) and collective responsibility, which is presumed to be high in a military population (20).

Whilst noting the limitations above, this study had a large number of responses, which was the highest in a military population (23–25). The study was also conducted at pace, when considering the rapid UK vaccination programme roll out and the loosening of COVID-19 restrictions as a result in decreasing numbers of infections and vaccination uptake (10). This study also further confirmed the literature that female sex and BAME ethnicity were associated with increased COVID-19 seriousness score (13, 14). The study also adds to the literature, by having one of the largest cohorts of young white Caucasian males sampled, with regards to their knowledge and attitudes towards COVID-19 preventative measures and vaccine uptake.

We consider there are two main benefits of this study. Firstly, it provided workforce-specific information, informing key RN policy makers and facilitating targeted information and educational campaigns for particular groups. Secondly, it shows that minor modifications to a validated knowledge and attitudes questionnaire can be quickly deployed and rapidly validated for other infectious diseases, increasing its potential future utility.

## Data availability statement

The raw data supporting the conclusions of this article will be made available by the authors, without undue reservation.

## Ethics statement

This study involving human participants were reviewed and approved by Ministry of Defence Research Ethics Committee. The patients/participants provided their written informed consent to participate in this study.

## Author contributions

SW, RC, AL, PM, MT, TF, and MO'S all contributed to the study design and manuscript preparation. SW, RC, AL, and PM contributed to the data collection. SW, RC, and JB contributed to the data analysis. JB contributed to the statistical analysis. All authors contributed to the article and approved the submitted version.
