# Supplementary material for: COVID-19 risk, attitudes and behaviour study (CRAB study): A knowledge, attitudes, and practise qualitative study of COVID-19 in the Royal Navy
Source: Front Public Health. 2023 Jan 12;10:1101817. doi: 10.3389/fpubh.2022.1101817 (PMC9878343; doi:10.3389/fpubh.2022.1101817)
Supplement: Supplementary file 1 [file Table_1.DOCX]

Supplementary Appendix

CRAB Study Questionnaire

Demographic information

1. What is your age?

- 18-24
- 25-34
- 35-44
- 45-54
- 55+

2. Which of the following best describes your biological sex?

- Female
- Male

3. Which of the following best describes your ethnic background?

- White - English, Welsh, Scottish, Northern Irish or British
- White - Irish
- White - Gypsy or Irish Traveller
- White - Any other White background
- Mixed or Multiple ethnic groups - White and Black Caribbean
- Mixed or Multiple ethnic groups - White and Black African
- Mixed or Multiple ethnic groups - White and Asian
- Mixed or Multiple ethnic groups - Any other Mixed or Multiple ethnic background
- Asian or Asian British - Indian
- Asian or Asian British -Pakistani
- Asian or Asian British - Bangladeshi
- Asian or Asian British - Chinese
- Asian or Asian British - Any other Asian background
- Black, African, Caribbean or Black British – African
- Black, African, Caribbean or Black British - Caribbean
- Black, African, Caribbean or Black British - Any other Black, African or Caribbean background
- Black, African, Caribbean or Black British -
- Other ethnic group - Arab
- Any other ethnic group
- Prefer not to say

4. What is your rank?

- AB-LH/Mne-Cpl (OR1-OR4)
- PO-CPO/Sgt-CSGT/WO2-WO1 (OR6-OR9)
- OC-SLt - Lt Cdr (RN)/2Lt-Lt - Maj (RM) (OF(D)-OF3)
- Cdr (RN)/ Lt Col (RM) and above (OF4+)

5. Which of the options below best defines your role/branch?

- Warfare
- Royal Marines
- Logistics
- Medical
- Engineer
- Air Crew
- Other

6. Which of the following options best describes your unit?

- Submarine
- FF/DD
- QEC/LPD
- Commando Unit
- Training Unit (Trainee)
- Training Unit (Instructor)
- MCMV/B2 OPV
- Air Station/Shore Establishment

7. Which of the following best describes your highest level of education?

- GCSEs - any grade (or equivalent)
- A/AS level (or equivalent)
- Bachelor's Degree with/without honours (or equivalent) or above
- Other

**Knowledge of COVID-19**

This section is intended to establish your knowledge of COVID-19. Read the statements below and indicate whether you think they are correct/incorrect, or you don’t know.

8. COVID-19 only affects the lungs

- Correct
- Incorrect
- Don't Know

9. COVID-19 always gives you symptoms

- Correct
- Incorrect
- Don't know

10. COVID-19 can only be contracted once in a lifetime

- Correct
- Incorrect
- Don't know

11. There is a vaccine that offers protection from COVID-19

- Correct
- Incorrect
- Don't know

12. COVID-19 can be prevented by good hygiene

- Correct
- Incorrect
- Don't Know

13. How serious do you think being infected with influenza is? (1 being not at all serious and 5 being extremely serious).

- 1 (Not at all serious)
- 2
- 3
- 4
- 5
- 6 (Extremely serious)

14. How serious do you think being infected with COVID-19 is? (1 being not at all serious and 5 being extremely serious).

- 1 (Not at all serious)
- 2
- 3
- 4
- 5
- 6 (Extremely serious)

15. How serious do you think meningitis is? (1 being not at all serious and 5 being extremely serious).

- 1 (Not at all serious)
- 2
- 3
- 4
- 5
- 6 (Extremely serious)

16. How likely do you think it is that you will contract influenza in the next 12 months?

- Very likely
- Somewhat likely
- Neither likely nor unlikely
- Somewhat unlikely
- Very unlikely

17. How likely do you think it is that you will contract COVID-19 in the next 12 months?

- Very likely
- Somewhat likely
- Neither likely nor unlikely
- Somewhat unlikely
- Very unlikely

18. How likely do you think it is that you will develop meningitis in the next 12 months?

- Very likely
- Somewhat likely
- Neither likely or unlikely
- Somewhat unlikely
- Very unlikely

19. How concerned are you about becoming infected with COVID-19 in the next 12 months?

- Very concerned
- Somewhat concerned
- Neither concerned nor unconcerned
- Somewhat unconcerned
- Not at all concerned

**Attitudes to COVID-19 preventative measures**

This section is intended to ascertain your attitudes towards the common measures in place to combat COVID-19.

20. If you take no preventative measures, rate how likely you feel it will be that you will get COVID-19 in the next 12 months? (whether or not you have received the COVID-19 vaccination).

- Highly unlikely
- Unlikely
- Possible
- Likely
- Highly likely

21. If you **were not** to be vaccinated against COVID-19, what would you think your likelihood of getting COVID-19 in the next 12 months is?

- Highly unlikely
- Unlikely
- Possible
- Likely
- Highly likely

22. If you **were** to be vaccinated against COVID-19, what would you think your likelihood of getting COVID-19 in the next 12 months would be?

- Highly unlikely
- Unlikely
- Possible
- Likely
- Highly likely

23. Do you feel that social distancing reduces the risk of getting COVID-19?

- Certainly not
- Probably not
- Maybe
- Probably yes
- Most certainly

24. Do you feel cleaning frequently touched surfaces significantly reduces the risk of getting COVID-19?

- Certainly not
- Probably not
- Maybe
- Probably yes
- Most certainly

25. Do you feel wearing face coverings reduces the risk of getting COVID-19?

- Certainly not
- Probably not
- Maybe
- Probably yes
- Most certainly

26. Do you feel frequent hand washing/use of alcohol gels reduces the risk of getting COVID-19?

- Certainly not
- Probably not
- Maybe
- Probably yes
- Most certainly

27. Do you feel isolation of cases/suspected cases reduces the risk of getting COVID-19?

- Certainly not
- Probably not
- Maybe
- Probably yes
- Most certainly

**Motivations to comply with disease control measures**

This section is intended to capture which factors affect compliance with measures to control COVID-19.

28. Which of the two factors listed below motivate you the most to take measures to reduce the risk of COVID-19?

- I am worried about being ill
- I want to protect my family
- I want to protect my colleagues
- I don't want to negatively affect the functioning of my unit
- Other:

29. From the factors below select those most likely to increase the likelihood of you not carrying out measures to reduce the risk of COVID-19?

- I am never ill
- COVID-19 is not serious
- I cannot transfer the virus if I have no symptoms
- The measures are too hard to follow fully
- People in my work environment don't follow the measures
- Workplace factors mean it is impossible
- I don't think they work
- I have been vaccinated
- I have frequent COVID-19 tests (i.e. at a LFD site)
- I believe I have had COVID-19
- Prefer not to say
- Other:

30. What factors might result in you breaking lockdown rules?

- Seeing family members
- Seeing friends
- Socialising with work colleagues
- Operational necessity
- Prefer not to say
- Other:

**Information requirements regarding COVID-19**

This section is intended to build a picture of what information you feel you require regarding COVID-19.

31. Which topics regarding COVID-19 would you want to receive information about?

- How COVID-19 is transmitted
- The incubation period of COVID-19
- Information about the vaccine
- Information about COVID-19 testing
- How I can reduce the risk in my work place
- How I can raise concerns about COVID-19 in my workplace
- How can I reduce the risk at home
- Other:

32. From whom would you like to receive this information? (pick 2 from the list below).

- Medical Centre
- Defence Public Health Unit
- Your Chain of Command
- Your immediate line manager
- Civilian Public Health Bodies (i.e. Public Health England, Health protection Scotland etc).
- Other:

**Attitude towards vaccination**

This is the final section of the survey. This section is intended to ascertain your feelings towards the COVID-19 vaccination.

33. Have you received a dose of a COVID-19 vaccination?

- Yes
- No

34.  If no to Q33- Do you intend to consent to be vaccinated against COVID-19?

- Yes
- No
- Maybe

35. Which sources of information do you trust the most regarding the COVID-19 vaccination?

- Medical Centre/Defence Medical Services
- Friends
- Family members
- Religious leaders
- Colleagues
- Chain of Command
- Social media shared/liked by friends
- Social media (any source)
- Mainstream media (i.e. BBC/ITV/UK news papers etc)
- Other:

**Supplementary appendix- Figure 1. Heat map of perceived seriousness of COVID-19 compared to influenza and meningitis in white males under the age of 35.** In white males under the age of 35 (n=52). The brighter squares represent the larger number of respondents per Likert score, with yellow squares representing a larger number than a darker colour.

**Supplementary appendix- Figure 2.** **Heat map of the perceived effectiveness of non-pharmaceutical interventions in white males (n=136) who were unsure or declining a COVID-19 vaccine.** The brighter squares represent the larger number of respondents per score, with yellow squares representing a larger number than a darker colour.
